# Oral health indicators and sociodemographic factors in Brazil from 2008 to 2015

**DOI:** 10.11606/s1518-8787.2021055002763

**Published:** 2021-05-06

**Authors:** Juliana Leandro dos Santos, Raquel Conceição Ferreira, Leonardo de Paula Amorim, Anna Rachel Soares Santos, Antônio Paulo Gomes Chiari, Maria Inês Barreiros Senna

**Affiliations:** I Universidade Federal de Minas Gerais Faculdade de Odontologia Departamento de Odontologia Social e Preventiva Belo HorizonteMG Brasil Universidade Federal de Minas Gerais. Faculdade de Odontologia. Departamento de Odontologia Social e Preventiva. Belo Horizonte, MG, Brasil; II Universidade Federal de Minas Gerais Faculdade de Odontologia Departamento de Clínica, Patologia e Cirurgia Odontológicas Belo HorizonteMG Brasil Universidade Federal de Minas Gerais. Faculdade de Odontologia. Departamento de Clínica, Patologia e Cirurgia Odontológicas. Belo Horizonte, MG, Brasil

**Keywords:** Public Health Services, Health Services Research, Indicators of Health Services, Oral Health, Longitudinal studies

## Abstract

**OBJECTIVE:**

To evaluate the annual variation of oral health and primary care coverage, the tooth extraction ratio, and the average of supervised toothbrushing in Brazilian municipalities according to social development and population size.

**METHODS:**

Public secondary data were analyzed. The outcomes were health service indicators (oral health coverage, primary health care coverage, tooth extraction ratio, and average of supervised tooth brushing) estimated for all Brazilian municipalities annually from 2008 to 2015. Mixed-effect multilevel regression models with random intercept and slopes were fitted with a cross-interaction term to estimate the annual percent variation according to the Municipal Human Development Index (MHDI) and population size.

**RESULTS:**

Municipalities with low MHDI presented an annual increase in oral health and primary care coverage of 2.65% and 2.23%, respectively, which was significantly higher than municipalities with medium and high MHDI. Oral health and primary care coverage were 69.26% and 35.00% lower among municipalities with a large population. Municipalities with medium and high MHDI showed an annual decrease in tooth extractions of 5.15% and 5.02%, respectively. An annual decrease was observed in the average of supervised toothbrushing of 9.81% and 4.57% in municipalities with low and medium MHDI, respectively. The tooth extraction ratio was higher among larger municipalities; the relation is inverse for supervised toothbrushing.

**CONCLUSIONS:**

The access to primary care and oral health services increased in Brazil, while a decrease occurred in mutilating treatment and provision of preventive actions, with disparities among municipalities with different MHDI levels over time.

## INTRODUCTION

The implementation of the Family Health Program in 1994 and its consolidation by the Family Health Strategy and the National Primary Care policy has shifted the primary health care model in the Brazilian Unified Health System (SUS)^1^. In 2000, the Family Health Strategy incorporated oral health care in the Primary Health Care approach. The National Oral Health Policy was instituted in 2004, expanding health care coverage and increasing the access to health promotion and disease prevention actions, aiming to modify the traditional curative-rehabilitative model, based on the principles of health care equity, integrality, and universality^2^.

One of the National Oral Health Policy strategies for reorienting the oral health care model is monitoring the “impact of oral health actions through appropriate indicators”^3^. Indicators are measures that summarize information about the efficiency and effectiveness of health systems^4^. Over time, indicators may guide managers’ decision-making allowing the consolidation, reorganization, or qualification of actions^5^^,^^6^.

Brazilian public health services have been monitored and evaluated using indicators for access to care, delivery of services, problem-solving capacity, and continuity of care^7^. However, a few oral health care indicators, which have been modified over time, contain gaps that obstruct a more comprehensive assessment of the quality of actions and performance of services within the Primary Health Care scope of SUS^7^. Currently, oral health care access may be evaluated using oral health care coverage, which estimates the degree of ease or difficulty in obtaining care^8^. Whether based on health promotion and disease prevention or regular curative-rehabilitative care, the oral health care model can be evaluated by the tooth extraction ratio in relation to other treatments and the average of supervised toothbrushing^7^. The combined use of these indicators can provide a more accurate evaluation of oral health service performance over time.

The use of indicators also allows comparisons among different geographic, demographic, or socioeconomic contexts showing disparities that can guide managers’ actions. Previous studies evaluated indicators of access to oral health care (First Programmatic Dental Consultation) and provision of oral health care (proportion of total procedures, tooth extraction ratio, total supervised toothbrushing)^9^. Some of these studies have shown that indicators can vary within a period of time^10^^,^^11^. Other cross-sectional studies showed differences in oral health indicators according to the Human Development Index, size of the population, and other municipal characteristics^12^^,^^13^^,^^14^. In general, studies have shown improvements in oral health indicators, but with the persistence of socio-regional inequalities^5^^,^ ^6^^,^^12^^,^^13^.

The change in health indicators with time might suggest an increase or a reduction in disparities among diverse contexts due to differences in investments and health policies. Therefore, the comparison of annual variations of health indicators according to municipalities’ socioeconomic and demographic profiles can lead to more equitable policies^5^^,^^14^. The population size has been used to stratify homogeneous groups to analyze the performance of health services^15^. Besides, the Municipal Human Development Index, which summarizes a complex reality of a city in a single number, was previously associated with indicators of oral health care quality^12^ and thus can be used to compare Brazilian municipalities over time.

Therefore, the research questions of this study were: 1) how did indicators of health services change in seven years? 2) Are these variations constant in municipalities with different MHDI and population size? 3) If not, how do MHDI and population size influence the variations? The present study aimed to estimate the annual variation of the primary health care and oral health coverage, the tooth extraction ratio, and the average of supervised toothbrushing from 2008 to 2015 and compare this variation in Brazilian municipalities with different MHDI and population size.

## METHODS

This was an ecological study with longitudinal data from 2008 to 2015 for all Brazilian municipalities.

### Outcomes

Four indicators were selected to evaluate the population’s access to primary oral health services (primary care coverage, oral health care coverage) and the offered care model (tooth extraction ratio and average supervised toothbrushin).

The primary care coverage and oral health care coverage indicators vary from 0 to 100, and higher values indicate a more significant offer of services to the population. The tooth extraction ratio is calculated by the ratio between tooth extractions and the total treatments performed (preventive and curative). Smaller ratios indicate a more conservative care model. The average of supervised toothbrushing coverage is the ratio between the number of people who participated in supervised collective toothbrushing and the total population. It measures access to preventive actions for dental caries and periodontal diseases.

These indicators were accessed through the TABNET link (Health Municipal Indicators – Guidelines, Aims and Goals, 2015) in the Department of Information Technology of the Unified Health System (DATASUS) website for each municipality in the 26 Brazilian states and Federal District for each year from 2008 to 2015. This period was selected because it had the complete data for the indicators of interest.

### Sociodemographic Profile of Municipalities

The variables selected to group municipalities according to the sociodemographic profile were MHDI and population size.

The HDI scores of each municipality in 2010 were retrieved from the *Atlas Brasil* website^16^. The scores are adapted from the country’s overall HDI by the United Nations Development Program in Brazil, the Institute for Applied Economic Research, and the João Pinheiro Foundation. The resulting index, termed the Municipal HDI (MHDI), has the same interpretation as the overall HDI, but at the municipal level. The MHDI encompasses three components (longevity, education, and income) with values ranging from 0 to 1, and an MHDI closer to 1 indicates a higher human development. The *Atlas Brasil* classifies the MHDI into “Very Low” (from 0 to 0.499), “Low” (from 0.500 to 0.599), “Medium” (from 0.600 to 0.699), “High” (from 0.700 to 0.799), and “Very High “(0.800 to 0.899) Municipal Human Development^16^. The municipalities were grouped into low MHDI (very low + low), medium, and high MHD I (high + very high) for analysis.

Brazilian municipalities were also stratified by population size according to the Brazilian Institute of Geography and Statistics classification from 2013 as follows: up to 5,000; from 5 to 9.9 thousand; from 10 to 49.9 thousand; from 50 to 100 thousand; > 100 thousand inhabitants. For the regression model, the two last categories were grouped^17^.

### Statistical Analysis

A descriptive analysis was performed, and mean values and respective 95% confidence intervals of each evaluated indicator for each year according to the MHDI and population size are displayed in graphs. The annual variation of the indicators was estimated using a regression model for longitudinal data (*xtmixed*) with unstructured covariance for each outcome. We fitted models with fixed effect, random intercept, or random intercept and slope, allowing for the effect of time on health indicators. The LR test was used to compare the models. A time cross interaction term was added to estimate the annual variation according to municipalities’ socioeconomic and demographic characteristics. We assessed the annual variation of indicators by groups, using marginal estimates, and we tested the significance of the difference in variations using the Wald test. The annual percent variation was obtained using the natural logarithmic transformation of the outcomes. Multicollinearity among HDMI and population size was measured by variance inflation factors (VIF) using the *collin* command from Stata^®^. VIF values close to 1.0 indicate that there is no problem with multicollinearity. The models were adjusted for the proportion of the population aged 60 or over, the proportion of women and rural residents in each municipality, and the fluoridation of public water supply. The first three covariates were obtained from the 2010 Brazilian census. The fluoridation of public water supply was based on information from the National Survey of Basic Sanitation carried out in 2008. The four covariates were obtained from the Institute of Geography and Statistics website. These covariates were chosen since they could affect the annual variation of health indicators due to different oral health profiles or access to oral health services. We used the statistical software Stata^®^ version 15.0 for all analyses.

## RESULTS

We found that out of the 5,570 Brazilian municipalities, 25.14% (n = 1399) had low MHDI and 40.13% (n = 2,233), medium. Regarding population size, the distribution was < 5,000 (n = 1,247, 22.39%), 5 to 9.9k (n = 1,226, 22.01%), 10 to 49.9k (n = 2,459, 44.15 %), 50 to 100k (n = 340, 6.10%) and > 100k inhabitants (n = 298, 5.35%). The data for oral health coverage, primary care coverage, and average of supervised toothbrushing was available for 99.89% of the municipalities. The tooth extraction ratio was recorded for 4925 municipalities over the period. The analysis included only municipalities with no missing data.

The municipalities with missing data for the tooth extraction ratio were not homogeneously distributed according to MHDI and population size. We observed missing data for at least one year of this indicator for 15.5% of the 1,399 municipalities with low MHDI, in 13.12% among the municipalities with medium HDI (n = 2,233), and 6.9% of 1,933 municipalities with high MHDI. For population size, the rates of municipalities with missing data were 15.24, 15.09, and 10.33% for municipalities with < 5k, 5 to 9.9k, and 10 to 49.90k inhabitants, respectively, and 4.12 and 0.67% for larger municipalities (50 to 99.9k and > 100k inhabitants, respectively).

**Figure 1:**
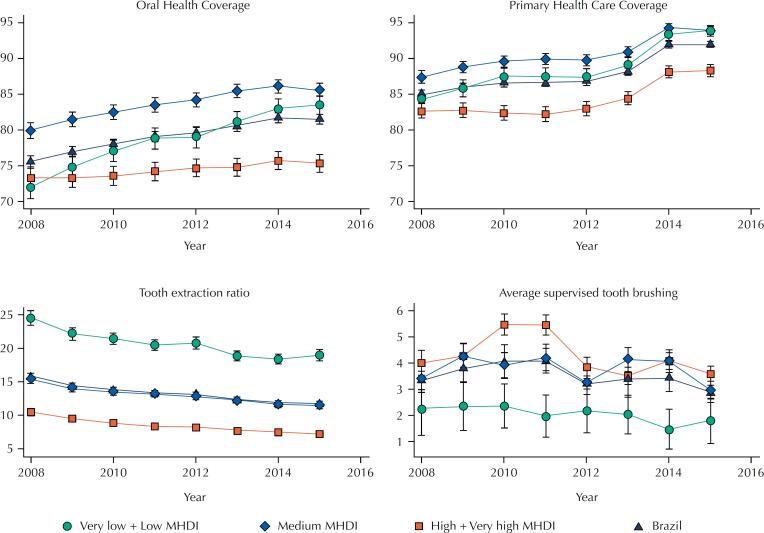
Mean and 95% confidence interval of primary health and oral health care coverage, tooth extraction ratio, and average of supervised toothbrushing from 2008 to 2015 according to the Municipal Human Development Index (MDHI) in Brazil.

Figure 1 shows the mean (confidence interval) of each indicator throughout the eight years according to MHDI. Municipalities with medium MHDI showed the highest mean coverage indicators throughout the period. The primary health care coverage was higher than oral health care coverage. There was a decrease in the tooth extraction ratio over time, which was higher among municipalities with low MHDI.

**Figure 2:**
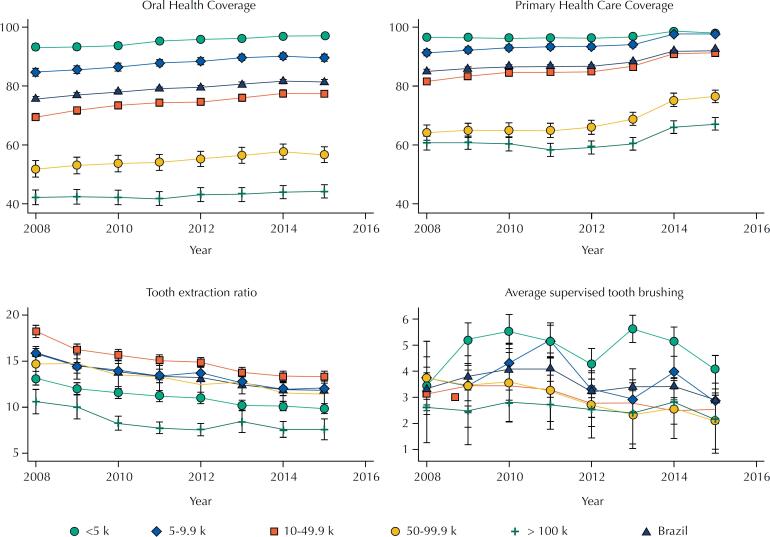
Mean and 95% confidence interval of primary health and oral health care coverage, tooth extraction ratio, and average of supervised toothbrushing from 2008 to 2015 according to population size in Brazil.

Figure 2 shows the mean of indicators according to population size over time. We observed higher oral health and primary health care coverage among municipalities with population < 5k. Municipalities with population > 100k presented lower oral health and primary health care coverage values and low tooth extraction ratio.

The HDMI and population size variables were not collinear (VIF < 1.44). The comparison of models considering fixed or random effects of time (year) was significant for all outcomes (LR test with p < 0.05). The cross-interaction term between year and MHDI was significant, showing that the annual variation was not fixed among municipalities with different MHDI levels. The interaction between population size and year was not significant. Then, the models were adjusted with an interaction term between time and MDHI. We included population size in the regression model to estimate the fixed effect of this variable. Table 1 shows the estimates of the model to log-transformed outcomes: primary care and oral health care coverage. The coefficient for time (year) is the effect of year on the indicator when MHDI is zero (low + very low). The interaction term indicates the effect of each year decreases primary care coverage by 0.0081 in municipalities with medium MHDI. The resulting coefficient was 0.0139, which we interpret as the annual percent variation of primary care coverage over time. Cities with low MHDI presented an annual increase of primary care coverage of 2.23%. This variation was significantly higher than those observed among municipalities with medium (1.39%) and high (1.31%) MHDI. There was no significant difference between the annual percent variation in primary care coverage among municipalities with high and medium MHDI (Table 1).

**Table 1:** Regression model and estimates of annual percent variation of oral health care coverage and primary care coverage and the difference among time variation according to Municipal Human Development Index (MHDI) from 2008 to 2015 in Brazil.

		Oral health care coverage	Primary care coverage
Year (time)		0.0265 (0.0234–0.0296)	0.0223 (0.0199–0.0244
MHDI			
	Medium	26.98 (19.13–34.82)	16.21 (101.47–232.99)
	High + very high	39.29 (31.24–47.34)	178.20 (11.32–243.92)
Population size			
	< 5 k		
	5–9.9 k	-0.0904 (-0.1211 to -0.0596)	-0.0098 (-0.0270–0.0075)
	10–49.9 k	-0.2823 (-0.3108 to -0.2538)	-0.0891 (-0.1051 to -0.0732)
	> 50 k	-0.6926 (-0.7344 to -0.6507)	-0.3500 (-0.3735 to -0.3266)
Interaction MHDI * year			
	Medium	-0.0134 (-0.0173 to -0.0095)	-0.0081 (-0.01112 to -0.0051)
	High + very high	-0.0195 (-0.0235 to -0.0155)	-0.0091 (-0.0122 to -0.0057)
	Intercept	-50.14 (-56.35 to -43.93)	-4039.95 (-445.90 to -364.01)
Random effects parameters			
	Variance (standard error) (year)	0.0026 (0.0001)	0.0013 (0.0001)
	Variance (standard error) (_cons)	1057.8 (254.84)	5477.37 (191.96)
	Covariance (standard error) (year,_cons)	-5.25 (0.13)	-2.72 (0.10)
Marginal estimates – Annual variation of the indicator (Y variation / time variation - DY/DT)			
	Low + very low MDHI	0.0265 (0.0248–0.0281)	0.0223 (0.01958–0.0243)
	Medium MDHI	0.0131 (0.0119–0.0145)	0.0139 (0.01196–0.0157)
	High + very high MDHI	0.0070 (0.0055–0.0084)	0.0131 (0.0111–0.0151)
Wald test – difference among slopes			
	Low + very low x medium	0.0265 (p < 0.001)	0.0081 (p < 0.001)
	Low + very low x high + very high	0.0131 (p < 0.001)	0.0091 (p < 0.001)
	Medium x high + very high	0.0069 (p < 0.001)	0.0004 (p = 0.861)

* The models were adjusted for the proportion of women, older adults, residents in rural areas, and fluoride presence in water supply.

The highest annual percent variation of oral health care coverage was observed in municipalities with low MHDI (2.65%). This variation was significantly higher than those found for municipalities with medium and high MHDI. In the period, the oral health and primary care coverage was 69.26% and 35.00% lower in larger municipalities (> 50k) compared to those with <5K, respectively (Table 1).

Municipalities with medium and high MHDI showed similar annual percent variations of tooth extraction ratio (5.15% and 5.02%, respectively). The annual average of supervised toothbrushing decreased by 9.81% and 4.57% among municipalities with low and medium MHDI, respectively (Table 2). We observed the lowest annual decrease among municipalities with high MHDI. Municipalities with 10 to 49.9 k inhabitants showed proportions of tooth extractions 20.30% higher than those with < 5k in the period. The average of supervised toothbrushing was 36.99, 77.94, 107.31% lower among municipalities with 5 to 9.9 k, 10 to 49.9 k, and > 50 k compared those with < 5 k.

**Table 2:** Regression model and estimates of annual percent variation of the tooth extraction ratio and the average of supervised toothbrushing and the difference in time variation according to the Municipal Human Development Index (MHDI) from 2008 to 2015 in Brazil.

		Tooth extraction ratio	Average of supervised toothbrushing
Year (time)		-0.0397 (-0.0479 to -0.0315)	-0.0981 (-0.1158 to -0.0800)
MHDI			
	Medium	23.35 (2.39–44.32)	-104.87 (-149.91 to -59.83)
	High + very high	20.33 (-1.09–41.76)	-152.89 (-198.98 to -106.80)
Population size			
	< 5 k		
	5–9.9 k	0.0982 (0.0450–0.1515)	-0.3699 (-0.4828 to -.2571)
	10–49.9 k	0.2030 (0.1539–0.2521)	-0.7794 (-0.8834 to -0.6755)
	> 50 k	0.1057 (0.0338–0.1777)	-1.0731 (-1.2246 to -0.9217
Interaction MHDI * year			
	Medium	-0.0118 (-0.0222 to - 0.0014)	0.0523 (0.0300–0.0748)
	High + very high	-0.0105 (-0.0212 to - 0.0002)	0.0764 (0.0539–0.0992)
	Intercept	82.50 (65.93–99.07)	196.95 (161.24–232.66)
Random effects parameters			
	Variance (standard error) (year)	0.0153 (0.0004)	0.0644 (0.0020)
	Variance (standard error) (_cons)	6204.22 (181.72)	26102.7 (813.6)
	Covariance (standard error) (year,_cons)	-30.85 (0.90)	-129.74 (4.04)
Marginal estimates – Annual variation of indicator (Y variation / time variation – DY/DT)			
	Low + very low MHDI	-0.0397 (-0.0479 to -.0315)	-0.0981 (-0.1158 to -0.0803)
	Medium MHDI	-0.0515 (-0.0579 to -0.0451)	-0.0457 (-0.0593 to -0.0320)
	High + very high MHDI	-0.0502 (-0.0569 to -0.0434)	-0.0217 (-0.0362 to -0.0072)
Wald test – difference among slopes			
	Low + very low x medium	0.0118 (p = 0.026)	-0.0524 (p < 0.001)
	Low + very low x high + very high	0.0105 (p = 0.053)	-0.0763 (p < 0.001)
	Medium x high + very high	-0.0013 (p = 0.781)	-0.0239 (p = 0.018)

* The models were adjusted for the proportion of women, older adults, residents in rural areas, and fluoride presence in water supply.

## DISCUSSION

The results showed an increase in primary care and oral health coverage and decreases in the tooth extraction ratio and supervised toothbrushing from 2008 to 2015, but with differences among municipalities according to MHDI.

Municipalities with a high MHDI had lower primary care and oral health coverage during the period compared to those with medium and low MDHI. Also, they showed the lowest annual percent increase in these indicators, independent of population size. The highest percent increase was observed in municipalities with low MHDI, which may have favored a decrease in differences in these indicators among municipalities with low and medium MHDI. However, the expansion of health care coverage in the municipalities with high MHDI is still a challenge, and the disparities are persistent over time. The lowest annual percent variation in primary care and oral health coverage among municipalities with high HDMI can result from fewer individuals who depend on the public health system, with a consequent slow expansion. These results reaffirm the previous findings of inequalities in access to oral health services^18^. The annual percent variation of primary health care and oral health coverage was constant for municipalities with different population sizes. During all the period, we observed lower coverage for larger municipalities. Although the results indicated an expansion of the population’s access to public health services in Brazil, they suggest it was insufficient to decrease disparities among municipalities.

The primary health care coverage was around 10% higher than oral health coverage in the evaluated years. The expansion of Primary Health Care coverage by including the Oral Health Teams in the Family Health Strategy was the first step for the oral health care consolidation in Brazil, given it is a central care provider that also organizes programs and projects. However, Oral Health Teams’ unequal insertion in the Family Health Strategy and the initial decision of having one Oral Health Team for every two Family Health Strategy centers seemed to have contributed to the gap between primary care and oral health care coverage. A previous study found that the closer to the ideal 1:1 ratio for oral health teams and family health strategy centers, the better the oral health indicators^14^. Thus, increasing the oral health care coverage at a similar rate as the Family Health Strategy coverage is a challenge and the goal for expanding access and improving the oral health indicators.

The highest annual percent variation in primary care and oral health care coverage in municipalities with medium and low MHDI suggests a “pro-equity” mechanism or even an “equity trend,” as there was a more significant expansion of services in the most vulnerable regions, which was also found in another study^18^. Since the Basic Operating Standard of SUS in 1996, there was a tendency to allocate the primary care budget according to the municipal MHDI to reduce social inequalities that impact health^19^. Also, as of 2004, there was an increase in financial support for the Family Health Strategy and Oral Health Teams in municipalities with low MHDI^20^. Thus, the results found in this study seem to be due to financing policies that favor equity in access to health care services in Brazil.

Similar to previous studies, larger cities had lower primary care and oral health care coverage^3^^,^^18^^,^^19^. This finding can be explained by the slower expansion of the Family Health Strategy in large urban centers^21^. Larger municipalities have a complex and diverse healthcare network that may financially compete with the structuring and consolidation of the Family Health Strategy^21^. Furthermore, problems related to the different operating conditions of the Family Health Strategy (infrastructure, inputs, equipment, and deficiencies in management and professional training) in big urban centers are coupled with the diversity of models for inserting the Family Health Strategy in primary health care.

The evaluation of tooth extraction rates is a strategy for monitoring oral health conditions and the public health care profile, generating useful information for planning and organizing oral health care^12^. We observed the highest tooth extraction ratio and the lowest annual percent variations in municipalities with low MHDI. The municipalities with medium and high MDHI presented similar annual reductions over the years. Demographic and socioeconomic conditions and access to oral health care services can determine tooth loss. SUS users seem to have worse oral health conditions than private care users, and often tooth extraction is the only treatment option. Municipalities with worse socioeconomic conditions tend to have greater difficulties in organizing and managing oral health services, resulting in less access, fewer preventive and restorative procedures, and less specialized care options, compromising the overall comprehensive care standard^4^. The difference in annual percent variation in coverage among municipalities may increase disparities, indicated by the high percentage of tooth extractions throughout the period in low MDHI cities.

The average of supervised toothbrushing is an indicator that replaced the Collective Procedures, which ran from 1992 to 2006 and remained in effect until 2012. These procedures were one of the strategies to reorganize the Oral Health Care model towards valuing collective and preventive actions for oral diseases and used as a mechanism for redistribution of wealth^21^. This indicator was also used for public dental services and care model performance^21^. We observed the highest average of supervised brushing in high MHDI municipalities of, which also presented an annual reduction of around 10% in the period. The smallest variation was observed in municipalities with low MDHI, which had the lowest average values of supervised brushing throughout the period. This result may be associated with lower availability of supplies for Oral Health Teams in more socially disadvantaged municipalities. Additionally, in general, municipalities with better socioeconomic conditions have better services and human resources and tend to offer more education, prevention, and health promotion actions^18^. The removal of the average of supervised toothbrushing from the Organizational Contract of Public Healthcare Action (COAP) from the year 2013^22^ explains the annual decrease in more developed municipalities. As this study used a collection of secondary data, there may be heterogeneity in data recording, which might have affected data quality. The analysis of missing data in the studied period showed a higher non-response rate among municipalities with low MHDI and smaller populations; being, therefore, more subject to estimation errors. This result indicates that socioeconomic disparities also affect the registration and information systems and the explanatory ability of indicators. Future studies should focus on monitoring the variations of oral health indicators given the recent changes in the National Primary Care policy and primary care financing^23^^–^^25^ and the consequences of such changes for access and quality of oral health care.

## CONCLUSION

From 2008 to 2015, Brazil expanded access to primary care and oral health services. We found a decrease in mutilating dental treatments and in the provision of preventive actions, with disparities among municipalities with different MHDI levels over time.
